# Distance-Dependent Plasmon-Enhanced Fluorescence of Submonolayer Rhodamine 6G by Gold Nanoparticles

**DOI:** 10.1186/s11671-021-03546-7

**Published:** 2021-05-22

**Authors:** Yajie Bian, Shikang Liu, Yuyi Zhang, Yiting Liu, Xiaoyu Yang, Shitao Lou, E. Wu, Botao Wu, Xiaolei Zhang, Qingyuan Jin

**Affiliations:** 1 State Key Laboratory of Precision Spectroscopy, East China Normal University, Shanghai, 200241 People’s Republic of China; 2 Collaborative Innovation Center of Extreme Optics, Shanxi University, Taiyuan, 030006 Shanxi People’s Republic of China

**Keywords:** Rhodamine 6G molecule, Gold nanoparticles, Poly (methyl methacrylate), Photoluminescence, Plasmon-enhanced fluorescence, Quenching effect, Atomic force microscope, Scanning tunneling microscope, Finite-difference time-domain method

## Abstract

We investigate the fluorescence from submonolayer rhodamine 6G molecules near gold nanoparticles (NPs) at a well-controlled poly (methyl methacrylate) (PMMA) interval thickness from 1.5 to 21 nm. The plasmonic resonance peaks of gold NPs are tuned from 530 to 580 nm by the PMMA spacer of different thicknesses. Then, due to the plasmonic resonant excitation enhancement, the emission intensity of rhodamine 6G molecules at 562 nm is found to be enhanced and shows a decline as the PMMA spacer thickness increases. The variation of spectral intensity simulated by finite-difference time-domain method is consistent with the experimental results. Moreover, the lifetime results show the combined effects to rhodamine 6G fluorescence, which include the quenching effect, the barrier effect of PMMA as spacer layer and the attenuation effect of PMMA films.

## Introduction

Fluorescence quenching [1–4] and enhancement [5–7] are two contradictory phenomena caused by the interaction between optical molecules and metals or metallic nanoparticles. In the past few decades, a considerable amount of reports published are focusing on the fluorophores emission properties in the near field of metal nanoparticles [8, 9]. These studies indicate that the suppression results from damped molecule dipole oscillation or orbital hybridization by the interface interaction [10–14], while the amplification is due to the highly enhanced incident field by local surface plasmon resonance [14–16].

Rhodamine 6G (R6G) is widely used as a fluorescent marker and laser dye for its stability, high fluorescence quantum efficiency and low cost. Most researches about R6G molecules have mostly focused on their solutions [17–20], while R6G molecules in solid state have been less studied [21, 22]. Meanwhile, although extensive researches have been carried out on the plasmon-assisted fluorescence, it is still too complicated to fully understand the interplay between the plasmonic resonance of metallic NPs and the intrinsic optical properties of molecules. In particular, matching the plasmonic resonance peak position with the emission peak of the fluorophore has been emphasized by many groups [23–26]. This is of great significance to understand the nature of plasmonic effects and for the development of molecular fluorescence-based measuring devices [27–31], such as organic light-emitting diodes (OLEDs) [32, 33], optical sensors [34, 35] and molecular electronic devices [36–38].

In our previous work, the single nanocrystal upconversion luminescence enhancement could be obtained by controlling surface plasmon resonance wavelength and NPs sizes [39]. The thickness of poly (methyl methacrylate) (PMMA) separating layer could be precisely controlled to tune the emission properties of single quantum dots [40]. Tetraphenyl porphyrin (TPP) molecules have been demonstrated to be affected dramatically by the localized plasmon mode [41, 42].

In this work, the plasmonic resonance peaks are tuned well overlapped with the molecular emission peak. The photoluminescence (PL) spectra and fluorescent lifetimes of submonolayer R6G molecules on the surface of gold NPs strengthen the evidence of a plasmon-enhanced dominance over nonradiative decay. This study provides an important opportunity to advance the understanding of single or submonolayer R6G molecules in solid state.

## Methods

### Fabrication of Substrate

To obtain the clean glass substrate with negative charges on its surface, the glass substrate was soaked in piranha solution for 30 min and rinsed by deionized water. Then, the glass substrate was put into 3 ml of 140-nm gold nanoparticle (Au NPs) solution (Crystano™) with a PH of 3.0 for over 12 h. Following this treatment, the Au NPs were absorbed firmly on the substrate based on electrostatic adsorption. After being washed and dried, the density of Au NPs on glass substrate was characterized by atomic force microscope (AFM).

Poly (methyl methacrylate) (PMMA) film was prepared by spin-coating at 3000 rpm for 60 s as a spacer between Au NPs and rhodamine 6G (R6G) molecules. For the purpose of controlling thicknesses of the spacer, PMMA methylbenzene solution with different concentrations of 0.03wt%, 0.1wt% and 0.4wt% was spin-coated on the glass surface.

### Submonolayer Rhodamine 6G Molecules Preparation

The submonolayer R6G molecules were sublimated to the surface of gold or glass substrate in 10^–6^ mbar vacuum at room temperature by thermal evaporation. The evaporation rate and the molecular coverage are controlled by continuous heating voltage, current and deposition time. The process was repeated several times using a scanning tunneling microscope (STM) in order to ascertain an appropriate preparation condition. The distribution of submonolayer R6G molecules on the substrates was characterized by STM and AFM.

### Photoluminescence

The photoluminescence (PL) spectra and fluorescence lifetime were obtained in 10^–5^ mbar vacuum at room temperature. Steady-state PL spectra were measured by a liquid nitrogen-cooled charge-coupled device (CCD) spectrometer (Princeton Instruments), while photon counting and lifetime measurements were completed with a microchannel plate photomultiplier tube (Hamamatsu) combined with time-correlated single photon counting technique (Edinburgh Instruments). A pulse picosecond semiconductor laser at 375 nm (Advance Laser System) was used to excite the samples.

### Simulation

The finite-difference time-domain (FDTD) method was used to perform the numerical simulation with the software FDTD Solutions (Lumerical Solution, Inc., Canada). Au nanoparticles with a diameter of 140 nm and different PMMA spacer thicknesses are placed on glass substrates. The dielectric constant of gold is taken from Ref. [43], and the dielectric constants of CTAB and PMMA are taken as 1.40 and1.49, respectively. The refractive index of the surrounding matrix is set to 1.0 for air. A plane-wave total field-scattered field source ranging from 400 to 700 nm is utilized as the incident light. The electric field distribution near Au nanoparticle is evaluated using the frequency-domain field profile monitors. A three-dimensional nonuniform meshing is used, and a grid size of 0.5 nm is chosen for the inside and vicinity of Au nanoparticle. We use perfectly matched layer absorption boundary conditions as well as symmetric boundary conditions to reduce the memory requirement and computational time. The numerical results pass prior convergence testing.

## Results and Discussion

**Fig. 1:**
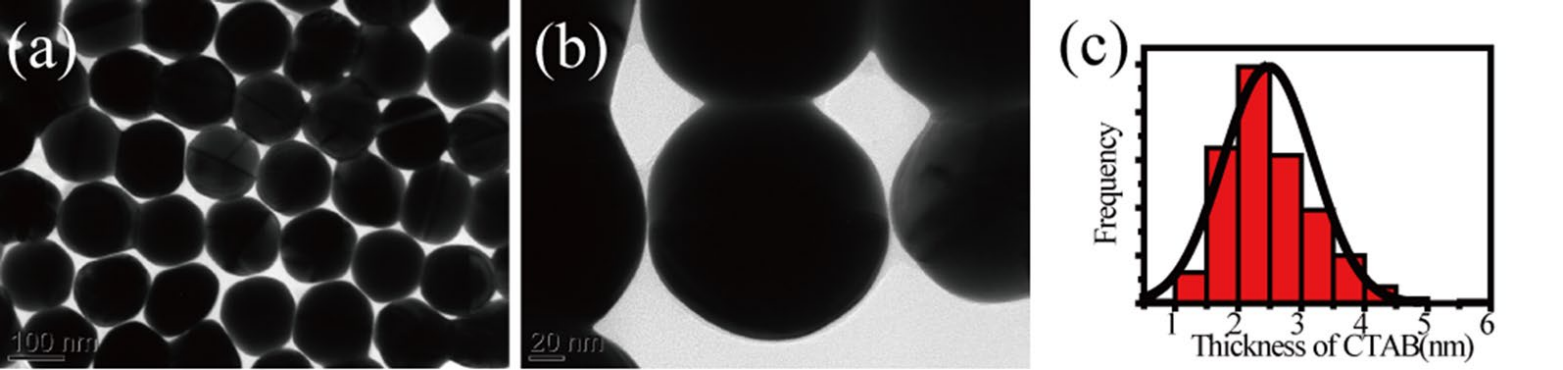
**a**, **b** TEM images of Au NPs with CTAB. **c** Thickness of CTAB distribution

The size and shape of 140 nm Au NPs were firstly characterized by a transmission electron microscope (TEM). As shown in Fig. 1a, most particles are well dispersed with an average diameter of 140 ± 10 nm. The gold NPs coated with ultrathin cetyltrimethylammonium bromide (CTAB) surfactant layers are also identified in Fig. 1b. The histogram shows that the shell thicknesses are 2.5 ± 1 nm (Fig. 1c), corresponding to monolayer or bilayer CTAB [44].

**Fig. 2:**
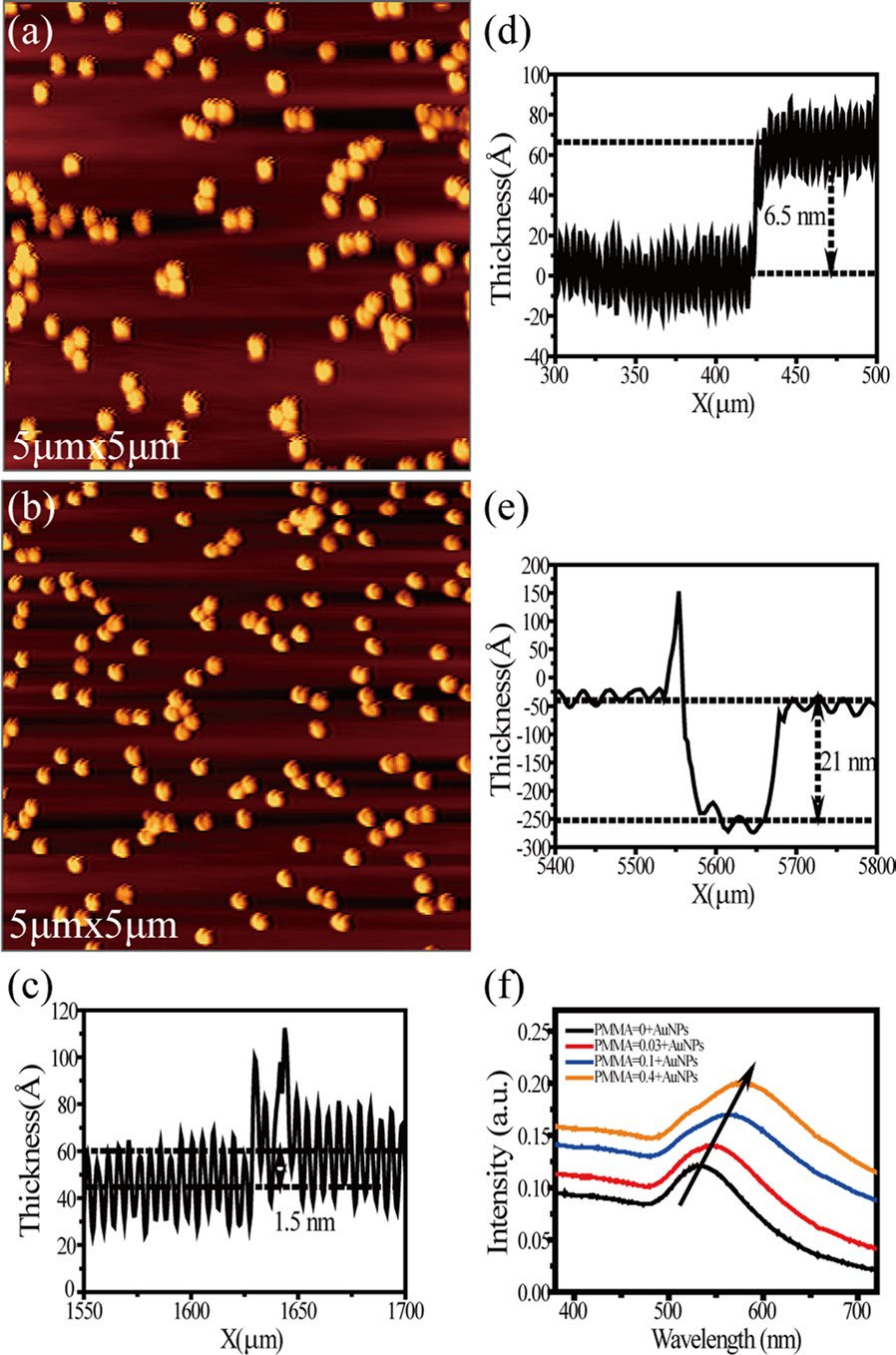
Typical AFM images of Au NPs/glass (**a**) and PMMA/Au NPs/glass (**b**). Surface profile of PMMA films with concentrations of 0.03wt% (**c**), 0.1wt% (**d**) and 0.4wt% (**e**). **f** UV–Vis absorption spectra of the NPs without PMMA (black) and with 1.5-nm (red), 6.5-nm (blue) and 21-nm (orange) PMMA separation layer

Typical AFM images of the Au NPs adsorbed on the glass substrate without and with a layer of PMMA are shown in Fig. 2a, b. Comparing Fig. 2a and b, we can find that the density of Au NPs in the same range is similar, but the spin-coated PMMA film cannot be distinguished by AFM image. Therefore, a benchtop stylus profilometer (Bruker) was used to assess the thickness of PMMA films with different concentrations. Figure 2c–e shows three samples with concentrations of 0.03wt% (~ 1.5 nm), 0.1wt% (~ 6.5 nm) and 0.4wt% (~ 21 nm), respectively. Figure 2f shows the absorption spectra of four different substrates. A clear redshift of surface plasmon resonance (SPR) peak can be found with the increasing thickness of PMMA layers. This might be due to the increase in the local refractive index around Au NPs, which has good consistency with the previous literature [45].

**Fig. 3:**
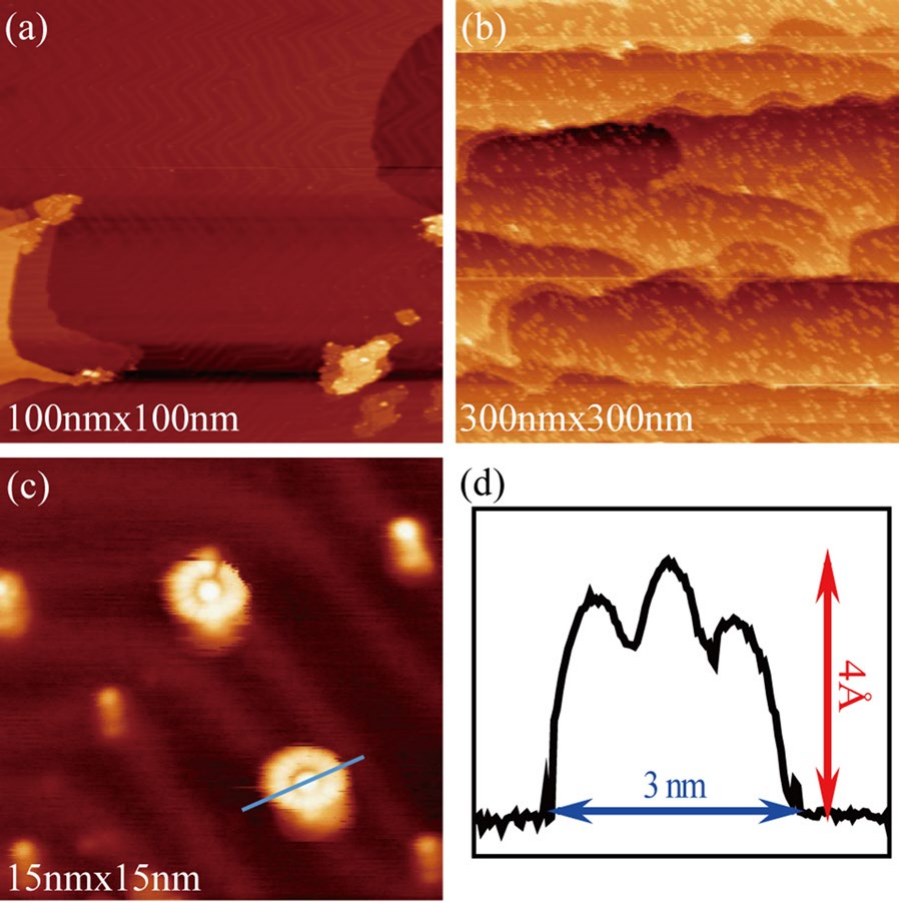
Typical STM image of the clean Au (111) (**a**), the R6G molecules on Au (111) (**b**) and a single R6G molecular (**c**). **d** Line profile across a single R6G molecule

Submonolayer R6G molecules were achieved by the following steps. Firstly, the Au (111) surface reconstructed after ion sputtering and high temperature annealing, which can be confirmed by its regular ‘herringbone’ stripes (Fig. 3a). Then, after 60 s of thermal evaporation at a voltage of 0.8 V and a current of 0.6 A, R6G molecules were steamed onto the treated Au (111) surface and cooled to 80 K using liquid nitrogen. The distribution state of molecular submonolayer can be characterized by STM (Fig. 3b). When narrowing the range, single isolated molecules, 3 nm in diameter and 0.4 nm in height, which are similar to poached eggs, can be observed stably and repeatedly (Fig. 3c, d). This can be attributed to weak molecular–substrate interaction.

**Fig. 4:**
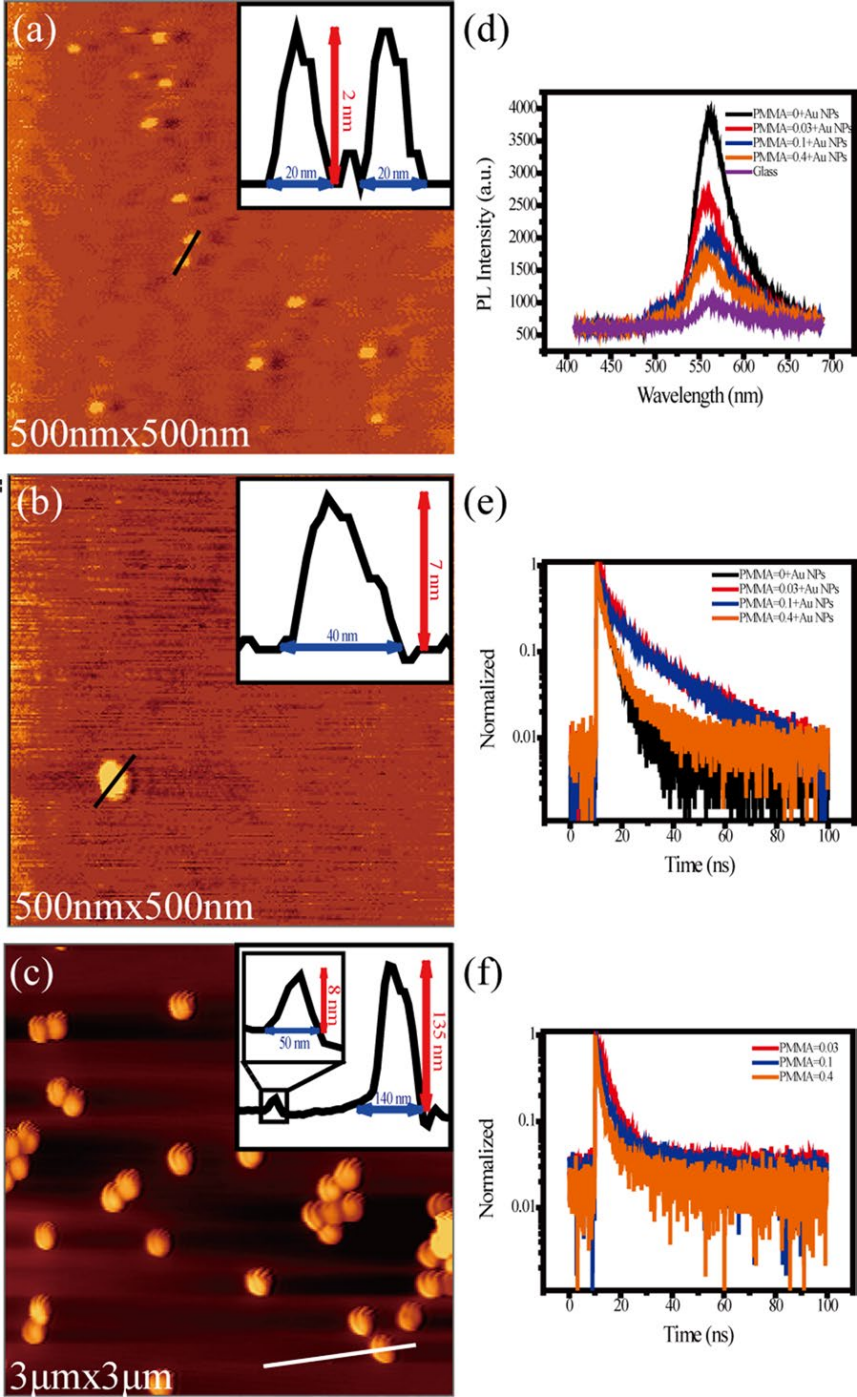
Typical AFM image of R6G molecules on glass (**a**), PMMA/glass (**b**), PMMA/Au NPs/glass (**c**). Insets: line profiles of R6G molecular clusters. **d**, **e** Fluorescence spectra and dynamics of PL decay of R6G/glass, R6G/Au NPs/glass and R6G/PMMA/Au NPs/glass, respectively. **f** Dynamics of PL decay of R6G/PMMA/glass

In the same condition, R6G molecules were deposited on the glass and PMMA/glass substrates, respectively. However, the room temperature is much higher than the temperature in the operation cavity of STM. So, the decrease of molecular–substrate interaction and the acceleration of molecular mobility make R6G molecules on the surface of the glass molecular clusters. But their coverage is still less than a single layer (Fig. 4a), which can also be verified on PMMA film (Fig. 4b). Comparing the insets of Fig. 4a and b, we can find that the size of the molecular clusters on the PMMA film is larger at room temperature in air, while the quantity decreases. This result may be explained by the fact that the migration rate and adsorption capacity of R6G molecules on different substrate surfaces are different.

Figure 4c shows the AFM image of R6G molecules on PMMA/Au NPs/glass. Due to the large difference in size between R6G molecular clusters and gold nanoparticles, it is difficult to observe both gold nanoparticles and molecular clusters at the same time. But the molecular clusters can be observed in the profile line (inset of Fig. 4c) compared with that on PMMA/glass.

Fluorescent spectra of R6G/PMMA/Au NPs/glass with different thicknesses of PMMA layers, R6G/Au NPs/glass and R6G/glass are plotted in Fig. 4d. The luminescent intensity of R6G/(PMMA)/Au NPs/glass is found to be enhanced compared with that of R6G/glass. From the luminescent enhancement factor of Table 1, we can see that R6G/Au NPs/glass has the largest enhancement with a factor of about 3.78. And its intensity decreases with the increasing thickness of the PMMA film.

**Table 1:** Lifetime and enhancement factor of the R6G/glass, R6G/Au NPs/glass and R6G/PMMA/Au NPs/glass samples

Sample	Density of PMMA (wt%)	Enhancement factor	*τ*_1_ (ns), *A*_1_	*τ*_2_ (ns), *A*_2_	*τ*_3_ (ns), *A*_3_
R6G/Au NPs/glass	**–**	3.78	0.42, 4	2.66, 67	5.68, 29
R6G/PMMA/Au NPs/glass	0.03	2.6	2.43, 11	5.26, 34	22.89,55
	0.1	2.01	0.62, 5	4.89, 30	22.39, 65
	0.4	1.67	0.55, 10	3.07, 62	13.09, 28
R6G/glass	**–**	1	–	**–**	**–**

Considering the absorption peaks of Au NPs coated with different thicknesses of PMMA (530–580 nm) and the emission peak of R6G molecules (562 nm), the fluorescence enhancement mechanism is related to the spectral overlap extent and the separation distance between molecules and nanoparticles. Both PMMA and CTAB on the surface of the gold sphere as the separation layers play key roles in reducing the nonradiative energy transfer between R6G molecules and Au NPs. Since plasmon resonance is a strong local near-field effect, the thickening of the interval layer makes R6G gradually get away from the range of strong local field. This leads to the weakening of the enhancement effect with the increase in PMMA thickness. On the other hand, the shift of the plasmon resonant peak also makes a contribution to the emission intensity. The absorption spectral linewidth of the four samples is very wide. All of them cover the emission peak of R6G. Although there is the best match between the plasmon resonant peak of Au nanospheres and the emission peak of R6G when the separation thickness is about 9 nm, the emission enhancement of R6G is not stronger than that without PMMA layer due to the decrease in near-field enhancement effect of Au nanospheres in large separation distance. Hence, the separation distance between Au nanospheres and R6G molecules plays a key role in the emission enhancement of R6G molecules.

In addition to the intensity change, the fluorescence lifetimes of R6G molecules are also detected, as shown in Fig. 4e. A tri-exponential function can well fit the decay process of excited R6G molecules, which are shown in Table 1. As the diagram shows, when the R6G molecule is directly evaporated onto the gold nanoparticles, the fluorescence lifetime is the shortest due to the quenching effect of the metal. With the thickening of the spacer layer, the quenching effect decreases, and the plasmonic enhancement effect also weakens, leading to the increase in the lifetime. However, the test results show that the fluorescence lifetime is not prolonged with the increasing thickness of PMMA, but gets shortened. Although, they are still longer than the molecular lifetime directly on the gold nanoparticles. In order to find out the reason, we tested the fluorescence lifetime of the R6G molecules on the PMMA/glass substrate (Fig. 4f and Table 2). It is also found that PMMA can affect the lifetime of the molecules, which decreases with increasing thickness. This is consistent with the phenomena of PMMA/Au NPs/glass. Therefore, the lifetime of the molecules in Fig. 4f is extended and then shortened, which results from the existence of PMMA film. When R6G molecules are close to Au NPs, the lifetime shows an obvious quenching effect. As the thickness of PMMA film increases, the barrier and the attenuation effects of PMMA film are observed.

**Table 2:** Lifetime R6G/PMMA/glass samples

Sample	Density of PMMA (wt%)	*τ*_1_ (ns), *A*_1_	*τ*_2_ (ns), *A*_2_	*τ*_3_ (ns), *A*_3_
R6G/PMMA/glass	0.03	1.22, 17	4.35, 72	18.29, 11
	0.1	0.67, 17	3.10, 50	19.00, 33
	0.4	0.57, 35	2.52, 35	14.77, 30

**Fig. 5:**
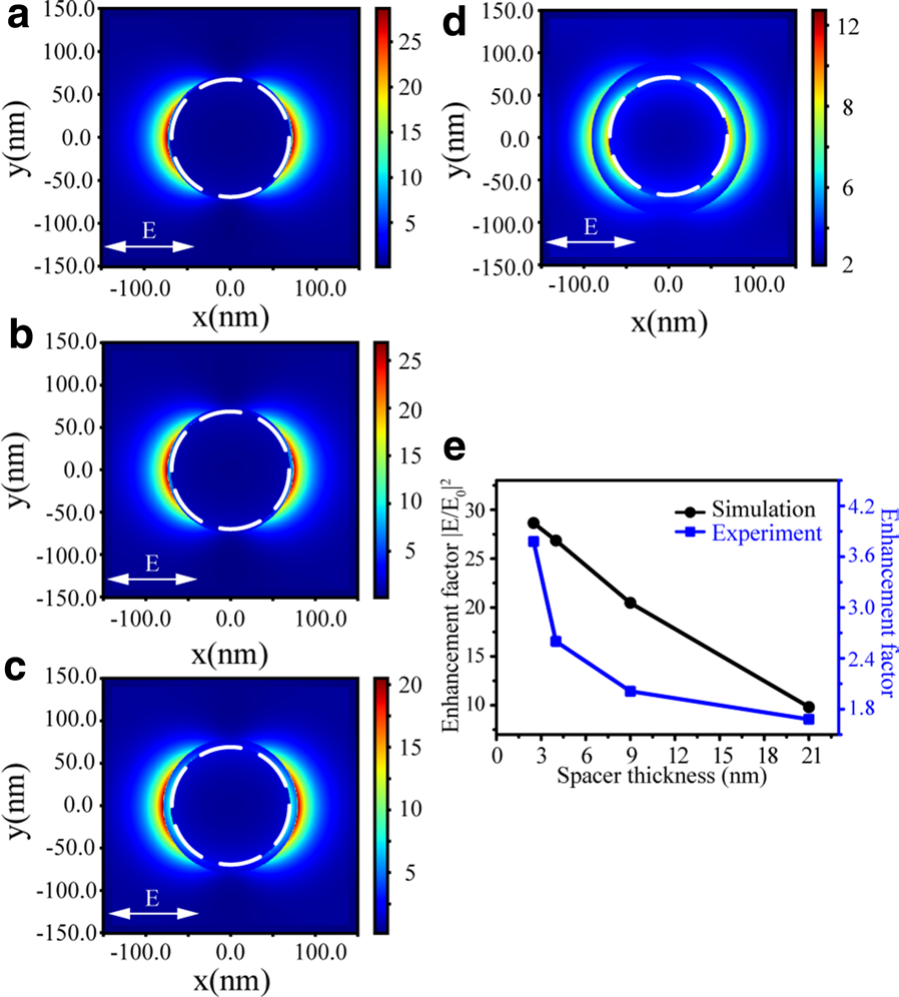
Electric field enhancement (|*E*/*E*_0_|^2^) distribution images for Au NPs covered with PMMA at *λ* = 562 nm with the spacer thickness 2.5 nm (**a**), 4 nm (**b**), 9 nm (**c**) and 21 nm (**d**), while the dashed white circle represents Au NP. **e** The enhancement factors of the experimental fluorescence spectra (blue) and the simulated electric field (black) dependent on the PMMA spacer thickness

To explain the observed distance-dependent fluorescence intensity of R6G molecules, the near-field distributions of Au NPs with different spacer thicknesses were simulated with the FDTD method. As shown in Fig. 5a–d, strong electric field enhancements (|*E*/*E*_0_|^2^) are observed around the surfaces of PMMA/CTAB/Au NPs nanostructures. In Fig. 5e, the enhancement factors of the experimental fluorescence spectra and the simulated electric field with the increasing spacer thickness display a good agreement. The simulated near-field enhancement factor is much larger than that obtained in the experiment. This reason can be mainly ascribed to the ideal models of theoretical simulations and the fluorescence quenching effect of Au NPs.

## Conclusion

In summary, we evaporated submonolayer R6G molecules on the gold NPs with the controlled PMMA spacer thickness (1.5–21 nm). The PL spectra and decay curves were studied. The molecular fluorescence intensity is enhanced by the resonant excitation enhancement and shows a decline as the thickness of PMMA film increased. The experimental enhancement factor is far below the theoretical one obtained by FDTD simulation mainly due to the quenching effect induced by the charge transfer and nonradiative energy transfer between the excited molecules and the Au NPs. Furthermore, it is interesting to note that the PMMA films with different thicknesses contain both barrier and lifetime attenuation effects, which is confirmed by the fluorescence lifetime measurements. This study may pave the way to the practical metal-enhanced fluorescence applications in optical imaging, biotechnology and material detection fields.

## Data Availability

The datasets used and/or analyzed during the current study are available from the corresponding author on reasonable request.

## Abbreviations

R6G: Rhodamine 6G
NPs: Nanoparticles
Au NPs: Gold nanoparticle
PMMA: Poly (methyl methacrylate)
OLEDs: Organic light-emitting diodes
TPP: Tetraphenyl porphyrin
PL: Photoluminescence
AFM: Atomic force microscope
STM: Scanning tunneling microscope
CCD: Charge-coupled device
FDTD: Finite difference time domain
TEM: Transmission electron microscope
CTAB: Cetyltrimethylammonium bromide
SPR: Surface plasmon resonance
